# Cryopreservation of *Cavia porcellus* L. Spermatozoa: Microscopic Characterization and Viability

**DOI:** 10.3390/ani16203211

**Published:** 2026-10-13

**Authors:** Yañez M. Castañeda-Pastor, Ilse S. Cayo-Colca, Jorge L. Maicelo-Quintana, Cesar A. Maraví-Carmen, Hurley Abel Quispe-Ccasa, Yander M. Briceño-Mendoza

**Affiliations:** 1 Facultad de Ingeniería Zootecnista, Biotecnología, Agronegocios y Ciencia de Datos, Universidad Nacional Toribio Rodríguez de Mendoza de Amazonas, Chachapoyas 01001, Amazonas, Peru; yanpastor26@gmail.com (Y.M.C.-P.); icayo.fizab@untrm.edu.pe (I.S.C.-C.); jmaicelo@untrm.edu.pe (J.L.M.-Q.); cesar.maravi@untrm.edu.pe (C.A.M.-C.); 2 Instituto de Investigación en Ganadería y Biotecnología, Chachapoyas 01001, Amazonas, Peru; 3 Dirección de Investigación y Desarrollo Tecnológico, Instituto Nacional de Innovación Agraria, La Molina, Lima 15024, Peru; hurleyabelqc@gmail.com

**Keywords:** *Cavia porcellus*, epididymal spermatozoa, sperm cryopreservation, semen quality, refrigeration, freezing curve

## Abstract

Studies evaluating the effects of cryopreservation on epididymal spermatozoa of *Cavia porcellus* remain limited. This study compared the effects of rapid and slow freezing protocols on microscopic characteristics, morphometry, and morphometric correlations. Epididymal spermatozoa from twelve guinea pigs were diluted in OptiXcell^®^ extender with 25% egg yolk and equilibrated at 4 °C for 6 h. One aliquot was evaluated, while the other was cryopreserved using rapid (*n* = 5) or slow (*n* = 7) protocols. After thawing, motility, viability, plasma membrane functionality, acrosomal and chromatin integrity, and morphometry were assessed; cooling profiles were characterized using linear and cubic regression models. Cryopreservation reduced microscopic parameters compared with refrigerated samples (*p* < 0.05). Comparison showed no significant differences in motility, viability, acrosomal integrity, or plasma membrane function (*p* > 0.05), whereas chromatin integrity was higher after slow freezing (*p* = 0.03). Rapid freezing reduced acrosomal area, acrosomal perimeter, and head length, whereas slow freezing preserved morphometric variables and correlations, although correlation patterns should be interpreted as associations among morphometric traits rather than structural preservation. Both cooling curves were well described by linear and cubic models. Slow freezing was associated with better preservation of chromatin integrity and selected morphometric characteristics.

## Simple Summary

Guinea pigs are an important animal species in several South American countries, where they contribute to food production, rural livelihoods, and the conservation of valuable genetic resources. The long-term preservation of male reproductive cells through freezing can support breeding programs and help maintain genetic diversity. However, little information is available on how freezing affects the quality of guinea pig spermatozoa. In this study, we compared two freezing methods to evaluate their effects on sperm quality after storage in liquid nitrogen (−196 °C). The results showed that the freezing process reduced overall sperm quality after thawing, while slow freezing was associated with better preservation of selected sperm characteristics. In addition, changes in cell size and structure were observed after freezing, indicating that this process can influence important characteristics related to reproductive performance. These findings contribute to a better understanding of reproductive preservation in guinea pigs and may help improve future breeding and conservation strategies. Developing more effective preservation protocols could support the sustainable use and genetic management of this species.

## 1. Introduction

Guinea pigs are of considerable socioeconomic importance in Peru and other South American countries [1]. The increasing demand for guinea pig production has intensified the movement of breeding animals across different geographic regions. However, transporting breeding stock entails biosecurity risks and may lead to the loss of animals with high genetic value. In this context, reproductive biotechnologies aimed at the preservation and dissemination of genetic material have emerged as strategic tools to support genetic improvement programs and contribute to the conservation of animal genetic resources.

Although semen cryopreservation has become a well-established reproductive biotechnology in domestic species for germplasm preservation, reduction of inbreeding, and mitigation of pathogen transmission, its application in guinea pigs remains limited, and current cryopreservation protocols still require substantial optimization [2]. Guinea pig spermatozoa exhibit specific morphological and morphometric characteristics involving the head, midpiece, and tail, while their plasma membrane appears particularly susceptible to cooling and freezing. These processes can induce membrane and acrosomal damage, DNA alterations, and oxidative and biophysical changes associated with cryopreservation [3]. However, how these morphological characteristics respond to different freezing conditions remains poorly characterized.

Although several studies have investigated sperm cryopreservation in guinea pigs [4,5], most have focused primarily on conventional sperm quality parameters, particularly motility and viability. As a result, important gaps remain in our understanding of how different freezing rates affect sperm structural and functional integrity [2]. In particular, little is known about their influence on sperm morphometry, plasma membrane functionality, acrosomal integrity, chromatin stability, and the relationships among morphometric characteristics before and after cryopreservation [6].

Because cryopreservation can affect multiple sperm structures and functions beyond motility and sperm viability, a comprehensive assessment should include plasma membrane functionality, acrosomal integrity, chromatin stability, and sperm morphometry. Evaluating these complementary parameters may provide a broader characterization of cryoinjury in guinea pig spermatozoa [6].

A comparative evaluation of rapid and slow freezing curves is particularly relevant because the cooling rate directly influences the magnitude of the physical and osmotic stress experienced by spermatozoa during cryopreservation. The effects of contrasting freezing rates on the combined microscopic and morphometric characteristics of *C. porcellus* epididymal spermatozoa remain insufficiently characterized [7].

Based on these considerations, the present study aimed to evaluate the effects of rapid and slow cryopreservation protocols on the microscopic and morphometric characteristics of epididymal spermatozoa from *Cavia porcellus*. Sperm motility, viability, acrosomal integrity, plasma membrane functionality, chromatin stability, and sperm morphometry were evaluated, together with the relationships among morphometric characteristics before and after cryopreservation.

## 2. Materials and Methods

### 2.1. Location and Animals

Semen quality analyses were performed at the Animal Andrology Laboratory and the Laboratory of Infectious and Parasitic Diseases of Domestic Animals at the Universidad Nacional Toribio Rodríguez de Mendoza de Amazonas, located in Chachapoyas, Amazonas, Peru. The study utilized twelve clinically healthy male Peruvian guinea pigs, aged 90 ± 7 days (approximately four months) and weighing between 900 and 1200 g. The animals were sourced from a commercial farm in the district of Jazan, Bongara, Amazonas, Peru, and housed at the experimental facilities of the Chachapoyas Research Station.

### 2.2. Experimental Design

The study was designed to evaluate the effects of refrigeration and two cryopreservation protocols (slow and rapid freezing) on the microscopic and morphometric characteristics of epididymal spermatozoa obtained from guinea pigs. Upon arrival at the Research Station, the animals underwent a two-day acclimatization period and were provided with alfalfa forage and water ad libitum. Animal handling procedures adhered to the ARRIVE 2.0 (Animal Research: Reporting of In Vivo Experiments) guidelines [8] and the AVMA Guidelines for the Euthanasia of Animals [9]. The experimental protocol was reviewed and approved by the Institutional Research Ethics Committee (CIEI) of the Universidad Nacional Toribio Rodríguez de Mendoza de Amazonas (UNTRM), Peru (protocol code CIEI-N° 00001; approval date: 23 February 2026).

The study design considered the principles of Replacement, Reduction, and Refinement (3Rs). Replacement was considered during study planning; however, no non-animal alternative could provide the epididymal spermatozoa required to evaluate the specific cryopreservation outcomes investigated in this study. Reduction was addressed by limiting the number of animals used and by avoiding the euthanasia of additional animals solely to achieve numerical balance between the freezing groups. Refinement was addressed through the two-day acclimatization period, appropriate husbandry, standardized animal handling, and the use of an approved euthanasia procedure.

The twelve animals were euthanized specifically for the present study to obtain epididymal spermatozoa for the evaluation of the cryopreservation protocols. Twelve clinically healthy male guinea pigs were included in the study, with each animal considered the experimental unit. Epididymal sperm recovered from both epididymides of each animal were pooled to obtain a single sperm suspension. The entire suspension was refrigerated at 4 °C for six hours to allow equilibration. After equilibration, the sperm suspension was divided into two aliquots. One aliquot (*n* = 12) was immediately used to evaluate microscopic sperm characteristics under refrigerated conditions, whereas the second aliquot (*n* = 12) was loaded into 0.5 mL straws and processed using either the slow (*n* = 7) or rapid (*n* = 5) freezing protocol.

This decision was consistent with the Reduction principle of the 3Rs, as it avoided the unnecessary use of additional animals while allowing the experimental information obtained from the animals already included in the study to be used. Consequently, the final distribution comprised five animals in the rapid freezing group and seven animals in the slow freezing group.

No formal randomization procedure was used to allocate animals to the freezing protocols, and no a priori power analysis was performed to determine the sample size. The animals were processed sequentially during the experimental period, and all animals included in the study met the established inclusion criteria. Cryopreserved samples were stored in liquid nitrogen for three days before post-thaw microscopic evaluation.

### 2.3. Sample Collection

The use of epididymal spermatozoa allowed the study objectives to be addressed without introducing an additional semen-collection procedure. Epididymal spermatozoa were collected following the Guidelines for the Euthanasia of Animals: 2020 Edition [8]. Euthanasia was performed by stunning, followed by cervical dislocation and a transverse incision of the carotid arteries and jugular veins, allowing approximately 30 s of exsanguination. After confirming the absence of the palpebral reflex, both testes were excised from the scrotal sac, placed into labeled tubes, and maintained at 37 °C in an insulated container until processing.

In the laboratory, the caudal regions of both epididymides were sectioned in a Petri dish on a thermal plate set to 37 °C. The Petri dish contained 0.4 mL of pre-warmed Tris medium (3.028 g Tris base molecular grade, 1.7 g citric acid, and 1.25 g D-fructose dissolved in 100 mL of distilled water) [10]. Small incisions were made to facilitate sperm release into the medium [11], followed by the addition of an extra 0.6 mL of Tris medium. The sperm suspensions recovered from both epididymides were pooled, transferred into 1.5 mL microcentrifuge tubes, and maintained at 37 °C until further processing.

### 2.4. Refrigeration and Freezing

OptiXcell^®^ medium (IMV Technologies, L’Aigle, France) was supplemented with 25% egg yolk based on the findings of [12], in which OptiXcell^®^ showed favorable results among commercial extenders, whereas Tris supplemented with 25% egg yolk showed favorable results among the egg-yolk-based formulations. This combination was adopted as an experimental adaptation to incorporate the characteristics of both formulations. The supplemented medium was pre-warmed in a 37 °C water bath (Gemmy Corp., Taipei City, Taiwan) and gradually added to the recovered sperm suspension in Tris medium. Sperm concentration and motility were determined individually in fresh samples using a a Makler counting chamber under a phase-contrast microscope (CX31, OLYMPUS, Tokyo, Japan), connected to a computer-assisted sperm analysis (CASA) system (SCA^®^ 5.3.0.0, VET Edition, Microptic, Barcelona, Spain) equipped with a digital camera (acA780-75gc, BASLER, Ahrensburg, Germany). The initial sperm concentration was 133.14 ± 90.53 × 10^6^ spermatozoa/mL, ranging from 54.40 to 300.00 × 10^6^ spermatozoa/mL (*n* = 12). Based on the individual sperm concentration and motility, the required volumes of sperm suspension and extender were calculated for each sample to standardize the cryopreservation to 30 × 10^6^ motile spermatozoa per 0.5 mL straw. The volume of OptiXcell^®^ required for each sample was calculated as the difference between the total volume required for the assigned number of 0.5 mL straws and the volume of sperm suspension. The mixture was then refrigerated for six hours at 4 °C to allow sperm equilibration. After the equilibration period, the refrigerated sperm suspension was divided into two aliquots. One aliquot was immediately used to evaluate refrigerated sperm characteristics, whereas the second aliquot was loaded into 0.5 mL straws for cryopreservation according to the assigned freezing protocol.

The straws were placed on a rack positioned 5 cm above the surface of liquid nitrogen inside a polystyrene box. The same rack position was maintained for both freezing protocols. Two electrical resistance elements positioned at the base of the polystyrene box were connected to a power outlet through an extension cord, and different connection/disconnection times were applied to generate the rapid and slow cooling profiles. Temperature changes were recorded every 30 s using a Type K thermocouple (Omega, Engineering, Stamford, CT, USA) until the temperature reached −140 °C. Cooling profiles were recorded for all experimental replicates (rapid freezing, *n* = 5; slow freezing, *n* = 7). Samples assigned to the slow freezing protocol reached −140 °C within approximately 10–12 min, whereas samples assigned to the rapid freezing protocol reached the same temperature within approximately 3–4.5 min. Once −140 °C was attained, the straws were immersed in liquid nitrogen (−196 °C) and stored in a cryogenic tank. After three days of cryogenic storage, the straws were thawed by immersion in pre-warmed water at 37 °C for 30 s, after which post-thaw microscopic sperm characteristics were evaluated. After three days of cryogenic storage, the straws were thawed by immersion in pre-warmed water at 37 °C for 30 s, after which post-thaw microscopic sperm characteristics were evaluated.

### 2.5. Microscopic Evaluation

#### 2.5.1. Motility

A 5 μL aliquot of the sample was placed on a pre-warmed glass slide at 37 °C and covered with a coverslip. Sperm analysis was conducted using a computer-assisted sperm analysis (CASA) system, SCA^®^ (SCA ^®^ 5.3.0.0. Edición VET, Microptic, Barcelona, Spain), connected to a phase-contrast microscope equipped with a 10× objective (CX31, OLYMPUS, Tokyo, Japan) and a camera (acA780-75gc, Basler, Ahrensburg, Germany) [13]. The system quantified total motility, progressive motility, and non-progressive motility. Progressive motility was selected as the motility parameter reported and analyzed in the present study.

#### 2.5.2. Vitality

The eosin–nigrosin supravital stain was utilized to assess sperm viability. A 5 μL aliquot of the sample (50 × 10^6^/mL) was mixed with 5 μL of 5% eosin solution and 5 μL of 10% nigrosin solution on a slide pre-warmed to 37 °C, then smeared and allowed to air dry. Once dry, 200 cells were counted under a microscope with a 40× objective (CX31, OLYMPUS, Tokyo, Japan). Spermatozoa stained red were classified as non-viable, whereas those appearing bright white were considered viable [14].

#### 2.5.3. Membrane Functionality

The hypoosmotic swelling test (HOST) was performed using a 50 mOsm/L solution containing 0.0245 g/mL sodium citrate and 0.9 g/mL D-fructose. A 25 μL aliquot of the sample was incubated with 100 μL of hypoosmotic solution at 37 °C for five minutes in a water bath. To halt the reaction, 31 μL of fixative solution (1 mL of hypoosmotic solution plus 3 μL of 40% formaldehyde) was added. A 5 μL aliquot of the final mixture was then placed on a slide, covered with a coverslip, and observed under a 40× microscope (CX31, OLYMPUS, Tokyo, Japan). A minimum of 200 cells were counted, with sperm classified as HOST-positive (HOST+) if a curled tail was present, and HOST-negative (HOST−) if the tail remained straight [10].

#### 2.5.4. Acrosome Integrity

Coomassie Blue 0.22% staining was performed following the protocol described by [15]. The stain was prepared using 0.11 g of Brilliant Blue G-250 (Merck, KGaA, Darmstadt, Germany), 25 mL methanol, 5 mL glacial acetic acid, and 20 mL distilled water. A 5 μL sample was smeared onto a glass slide and air-dried. Once dry, the slides were horizontally fixed in 4% formaldehyde in PBS (prepared with 10 mL of 40% formaldehyde and 90 mL PBS) for 15 min, followed by five sequential 1 s washes in PBS. For staining, the slides were immersed in a Petri dish containing Coomassie Blue solution for 5 min, then washed in distilled water with five brief 1 s immersions before air-drying.

Microscopic examination was conducted under a 40× objective (CX31, OLYMPUS, Tokyo, Japan), with a minimum of 250 cells counted. Cells were categorized into three groups: intact acrosome (CB++, intense blue staining), expanding acrosome (CB+, light blue staining), and absent acrosome (CB−, no staining) [10].

#### 2.5.5. Chromatin Decondensation

A 5 μL aliquot of the sample was smeared onto a slide and air-dried. Once dry, the slide was horizontally immersed in 96% ethanol in a Petri dish for two minutes, then stained in toluidine blue solution (prepared with 1 mL toluidine blue and 9 mL buffer, pH 4) for five minutes. The slide was subsequently rinsed with distilled water and dried in a dark, controlled environment. A total of 300 sperm cells per sample were counted and classified under a 40× objective microscope (CX31, OLYMPUS, Tokyo, Japan), following the method described by Carretero [16].

### 2.6. Morphology

Air-dried smears were stained using the Hemacolor^®^ cell differentiation kit (Merck KGaA, Darmstadt, Germany). The staining process involved five 1 s dips in the fixative, three 1 s dips in eosin solution, six 1 s dips in methylene blue solution, and two 10 s dips in the buffer solution, followed by air drying.

Morphometric analysis was conducted using an Olympus BX53 microscope (OLYMPUS, Tokyo, Japan) equipped with a U-TV0.63XC camera (OLYMPUS, Tokyo, Japan) and cellSens Standard software, version 4.1 (OLYMPUS, Tokyo, Japan), with immersion oil under a 100× objective. A total of 100 spermatozoa were evaluated per sample. Spermatozoa were selected from different microscopic fields and included in the morphometric analysis only when the entire spermatozoon was clearly visible, with a well-focused head, an intact and fully visible tail, and without overlap with adjacent cells or debris. These selection criteria were applied consistently to all samples to ensure accurate and reproducible morphometric measurements. Digital images of the selected spermatozoa were captured to measure nuclear length, nuclear width, nuclear area, nuclear perimeter, acrosomal area and perimeter, head length, midpiece length and width, and tail length [13].

### 2.7. Characterization of Freezing Curves

To characterize the cooling profiles of the slow and rapid freezing protocols, temperature changes within the freezing container were monitored throughout the cryopreservation process using a Type K thermocouple (Omega Engineering, Stamford, CT, USA). The cooling profiles were generated using two electrical resistance elements positioned at the base of the polystyrene box. The resistance elements were connected to a power outlet through an extension cord, and different connection/disconnection times were applied to generate the rapid and slow cooling profiles. The rack supporting the straws was maintained at 5 cm above the liquid nitrogen surface for both protocols. Temperature readings were recorded manually at 30 s intervals from the beginning of cooling (4 °C) until the straws reached approximately −140 °C. Cooling profiles were recorded for all experimental replicates (rapid freezing, *n* = 5; slow freezing, *n* = 7). The cooling profiles were consistent among replicates within each freezing protocol, as reflected by the observed variation in cooling rates. The mean cooling rate was 35.98 ± 5.70 °C/min (range: 30.22–48.00 °C/min) for rapid freezing and 13.26 ± 1.13 °C/min (range: 12.00–14.40 °C/min) for slow freezing. Upon reaching approximately −140 °C, the straws were plunged into liquid nitrogen (−196 °C) for storage. Because the objective of the regression analysis was to characterize the typical cooling behavior of each protocol, one representative cooling profile from each freezing protocol was selected for graphical presentation and regression modeling. The relationship between temperature (°C) and time (min) was analyzed by fitting linear and cubic polynomial regression models. Cubic polynomial models were used to describe non-linear temperature changes during cooling, whereas linear regression was applied to estimate the average cooling rate when appropriate. Model fit was assessed using the coefficient of determination RStudio (R^2^), version 2026.05.1+225 (Posit Software, PBC, Boston, MA, USA). Regression analyses were performed using Minitab Statistical Software, version 21.2 (Minitab LLC, State College, PA, USA; 2023).

### 2.8. Statistical Analysis

Statistical analyses were performed using R software (version 4.6.0; R Foundation for Statistical Computing, Vienna, Austria). The individual animal was considered the experimental unit. Data are presented as mean ± standard deviation (SD). For each variable, normality was assessed separately using the Shapiro–Wilk test, whereas homogeneity of variances between independent groups was evaluated using Levene’s test. Comparisons between the slow and rapid freezing protocols were performed using Welch’s independent-samples *t*-test. Comparisons between refrigerated and cryopreserved semen obtained from the same animal were analyzed using the paired Student’s *t*-test when data followed a normal distribution or the Wilcoxon signed-rank test when the normality assumption was not met. Effect sizes were calculated using Cohen’s *d*z for within-animal comparisons between pre-freezing and post-thaw measurements and Hedges’ *g* for the independent post-thaw comparison between the rapid and slow freezing groups. Ninety-five percent confidence intervals (95% CIs) were calculated for mean differences. Correlations among sperm morphometric variables were assessed using Spearman’s rank correlation coefficient. To control for multiple comparisons, *p*-values were adjusted using the Benjamini–Hochberg false discovery rate (FDR) procedure. Cooling profiles were characterized by fitting linear and cubic polynomial regression models relating temperature (°C) to time (min). Model performance was evaluated using the coefficient of determination (R^2^). Statistical significance was established at *p* < 0.05.

## 3. Results

### 3.1. Effect of Rapid and Slow Freezing Protocols on Microscopic Sperm Characteristics

Table 1 summarizes the microscopic characteristics of epididymal spermatozoa before freezing (after 6 h of refrigeration) and after thawing following rapid and slow freezing protocols. In both freezing protocols, progressive motility (PM), vitality, acrosomal integrity, and plasma membrane functionality (HOST+) were significantly reduced after thawing compared with the corresponding refrigerated samples (all *p* < 0.05). In contrast, chromatin integrity remained unchanged after slow freezing (*p* = 0.948) but decreased significantly after rapid freezing (*p* = 0.019). Direct post-thaw comparison between the rapid and slow freezing protocols showed no statistically significant differences in PM (*p* = 0.10), vitality (*p* = 0.65), acrosomal integrity (*p* = 0.38), or HOST+ (*p* = 0.10). In contrast, chromatin integrity was significantly higher after slow freezing than after rapid freezing (*p* = 0.03), with a mean difference of 7.13 percentage points (Hedges’ *g* = 1.28). Thus, among the microscopic sperm characteristics evaluated, the direct comparison between freezing protocols identified a statistically significant difference only for chromatin integrity.

### 3.2. Effect of Cryopreservation on Sperm Morphometric Characteristics

Table 2 summarizes the morphometric characteristics of epididymal spermatozoa before freezing and after thawing following the rapid and slow cryopreservation protocols. Rapid freezing significantly reduced acrosome area, acrosome perimeter, and head length compared with pre-freezing values (*p* < 0.05), whereas no significant changes were observed in nuclear dimensions, midpiece length, midpiece width, or tail length (*p* > 0.05). In contrast, the slow freezing protocol preserved nearly all morphometric characteristics after thawing, with a significant reduction detected only in midpiece width (*p* = 0.039). The within-protocol comparisons indicated fewer significant morphometric changes after slow freezing than after rapid freezing.

### 3.3. Correlation Analysis of Sperm Morphometry

#### 3.3.1. Rapid Freezing Protocol

Table 3 and Figure 1 present the Spearman correlation matrix between morphometric variables measured before cryopreservation (pre-freezing) and after thawing following the rapid freezing protocol. Overall, few significant correlations were observed between pre-freezing and post-thaw morphometric variables, whereas most associations were weak and not statistically significant. The correlation pattern differed markedly between pre-freezing and post-thaw measurements. Significant correlations were primarily observed among variables associated with the acrosomal region and head morphology, whereas tail length before freezing showed strong negative correlations with post-thaw nucleus width (ρ = −1.00, *p* < 0.001), acrosome area (ρ = −0.89, *p* < 0.05), and head length (ρ = −0.89, *p* < 0.05). In contrast, only a few positive correlations remained significant, particularly those between pre-freezing midpiece width and post-thaw nucleus length (ρ = 0.89, *p* < 0.05) and nucleus area (ρ = 0.89, *p* < 0.05).

#### 3.3.2. Slow Freezing Protocol

Table 4 and Figure 2 present the Spearman correlation matrix between pre-freezing and post-thaw sperm morphometric variables following the slow freezing protocol. Significant positive correlations were observed mainly among nuclear morphometric variables and between midpiece length and post-thaw midpiece width, with the correlation between pre-freezing midpiece length and post-thaw midpiece width showing the highest coefficient (ρ = 0.95, *p* < 0.001). Two significant negative correlations were detected, both involving pre-freezing acrosomal measurements and post-thaw nucleus width. These corresponded to acrosome area and nucleus width (ρ = −0.93, *p* < 0.01), as well as acrosome perimeter and nucleus width (ρ = −0.93, *p* < 0.01).

Overall, the slow freezing protocol maintained a greater number of significant positive correlations among morphometric variables than the rapid freezing protocol.

### 3.4. Cooling Curves During Cryopreservation of Cavia porcellus Spermatozoa

Figure 3 illustrates the cooling profiles obtained during the slow and rapid freezing protocols. Cubic polynomial regression models accurately described the non-linear cooling dynamics of both protocols (R^2^ = 0.99), whereas linear regression models were used to estimate the mean cooling rate over the entire freezing period (Table 5). The slow freezing protocol reached approximately −140 °C in about 12 min, corresponding to an average cooling rate of 11.25 °C/min (R^2^ = 0.95). In contrast, the rapid freezing protocol reached approximately −140 °C within about 4 min, corresponding to a substantially faster average cooling rate, although the linear model showed a lower goodness-of-fit (R^2^ = 0.74).

## 4. Discussion

Sperm motility is a key parameter for estimating the fertilizing potential of spermatozoa and remains one of the most important indicators in routine semen evaluation. In the present study, epididymal sperm from *C. porcellus* underwent a six-hour equilibration period at 4 °C before cryopreservation, resulting in progressive motility values ranging from 65.05% to 71.64%. These values were higher than those previously reported in guinea pigs, including 21.68% after three hours of refrigeration in a Tris-based extender supplemented with 25% egg yolk [12], 43.48% and 46.00% after two hours of refrigeration [10,17], and values ranging from 54.17% to 68.33% under similar storage conditions [18]. Refrigeration reduces the physiological temperature of spermatozoa, thereby decreasing metabolic activity and contributing to the preservation of sperm function [3].

Following thawing, sperm samples cryopreserved using the rapid and slow freezing protocols exhibited progressive motility values of 8.53% and 10.57%, respectively, with a numerical advantage observed for the slow freezing protocol. Although this difference was not statistically significant, the numerical advantage observed after slow freezing may be related to the more gradual temperature decline, which may allow progressive cellular dehydration and osmotic adaptation during cooling, thereby reducing abrupt membrane perturbations. However, this interpretation should be considered cautiously because progressive motility also differed significantly between the experimental groups before freezing, and therefore the post-thaw difference cannot be attributed exclusively to the freezing protocol. Reports on the cryopreservation of *C. porcellus* spermatozoa remain scarce. However, a recent study comparing conventional slow and ultra-rapid freezing in epididymal guinea pig spermatozoa reported higher post-thaw progressive and total motility, viability, and acrosomal and DNA integrity following ultra-rapid freezing than conventional slow freezing [5]. Previous studies have reported no progressive motility and only 0.64% non-progressive motility after thawing [12], whereas another study reported a post-thaw motility of 3.00% using glycerol as the cryoprotectant [18]. The contrasting results between these studies and the present findings may reflect differences in the specific freezing procedures used, including the cooling configuration, sample volume, cryoprotectant composition, and the cooling rate. In the recent study, ultra-rapid freezing was achieved by directly immersing 20 µL sperm droplets in liquid nitrogen, whereas the present study used defined rapid and slow cooling curves. Cryopreservation inevitably reduces sperm motility because osmotic stress, intracellular ice formation, membrane phase transitions, and oxidative damage impair the structural and functional integrity of spermatozoa [19]. However, the extent of this reduction depends on multiple factors, including semen source, extender composition, cryoprotectant type, cooling rate, freezing protocol and species-specific sperm characteristics.

In the present study, epididymal spermatozoa were cryopreserved in the absence of seminal plasma, which may play an important role in maintaining the viability and functionality of ejaculated spermatozoa. Therefore, direct comparisons between epididymal and ejaculated spermatozoa should be interpreted with caution, since the absence of seminal plasma may influence post-thaw sperm performance. Previous studies using semen collected by electroejaculation have reported substantially higher progressive motility values in fresh semen, ranging from 72.15% to 91.00% [10,18]. Although both experimental groups underwent the same equilibration period before freezing, the slow freezing protocol yielded numerically higher post-thaw progressive motility than the rapid freezing protocol. Previous studies have also shown that the equilibration period preceding freezing facilitates the adaptation of sperm membranes and intracellular organelles to the structural changes induced during cryopreservation [20,21].

Sperm viability is a fundamental parameter for assessing the success of cryopreservation procedures [4]. Following the six-hour equilibration period, refrigerated epididymal spermatozoa exhibited viability values ranging from 63.50% to 69.71%, which were comparable to the 64.24% previously reported using a Tris-based extender supplemented with 25% egg yolk [12]. Higher viability values, ranging from 72.65% to 86.70%, have been reported after refrigeration at 5 °C for three hours [13,22]. In contrast, lower viability values of 35.90% after two hours of refrigeration at 5 °C [17] and 34.86% in fresh epididymal spermatozoa [10] have also been described. After thawing, viability decreased to 15.70% following rapid freezing and 18.36% following slow freezing, with a numerical advantage observed for the latter protocol. The post-thaw viability achieved with the slow freezing protocol exceeded the values previously reported for two freezing protocols, which ranged from 14.42% to 15.08% [23], whereas the viability obtained with the rapid freezing protocol was comparable to those reports. Recent evidence in guinea pig epididymal spermatozoa also demonstrates that cryopreservation markedly reduces post-thaw sperm quality, while extender composition can influence the extent of membrane protection during freezing and thawing [24]. The decline in sperm viability may be related to cryopreservation and its detrimental effects on sperm structure, particularly membrane lipid phase transitions that occur during cooling [25]. A slower cooling rate may reduce abrupt changes in membrane organization by allowing more progressive cellular dehydration and adaptation to extracellular solute changes, thereby limiting membrane destabilization during cooling and thawing. These processes may help explain the numerical advantage in post-thaw viability observed after slow freezing in the present study. In addition, extender composition may influence post-thaw sperm survival, particularly if it does not fully meet the physiological requirements of guinea pig spermatozoa.

The acrosome is an essential structure involved in sperm hyperactivation, capacitation, and the acrosome reaction preceding fertilization [26]. Therefore, acrosomal integrity is considered a key indicator for assessing the efficiency of cryopreservation protocols. Owing to its high affinity for protein-rich structures, Coomassie Blue staining has been widely used to evaluate acrosomal status in spermatozoa, including those of camelid species [15]. The acrosome of *C. porcellus* is rich in enzymatic components [10] and spermatozoa with an intact acrosome are characterized by an intensely blue-stained acrosomal cap covering the anterior region of the nucleus (strong CB).

In the present study, acrosomal integrity was comparable between the two freezing protocols. Following the six-hour equilibration period, refrigerated sperm samples exhibited acrosomal integrity values ranging from 46.88% to 53.09%. After thawing, acrosomal integrity declined to 11.76% and 15.14% in the rapid and slow freezing protocols, respectively. Although both cryopreservation protocols substantially reduced acrosomal integrity, the slow freezing protocol yielded numerically higher post-thaw values. The high susceptibility of the guinea pig acrosome to cryoinjury may be associated with its distinctive structural characteristics. Compared with other domestic species, the acrosomal architecture of *C. porcellus* appears to be particularly vulnerable to the physical and osmotic stresses generated during cooling and freezing [26]. The numerical difference observed between protocols may be related to the rate at which spermatozoa undergo cellular dehydration and osmotic adjustment during cooling. A slower cooling rate may allow these changes to occur more progressively, potentially reducing abrupt volume changes and membrane stress in the acrosomal region. Previous studies reported higher proportions of strong Coomassie Blue staining in refrigerated spermatozoa (74.40%), but considerably lower values after thawing (5.27%) [12]. These differences among studies suggest that extender composition, cryoprotectant type, and cooling protocol may influence the preservation of acrosomal integrity.

The hypoosmotic swelling test (HOST) was used to evaluate plasma membrane functionality based on the sperm endosmosis response (HOST+) [27]. Following the six-hour equilibration period, HOST+ values ranged from 23.71% to 30.30%. After thawing, plasma membrane functionality decreased in both freezing protocols, with numerically lower HOST+ values observed in the rapid freezing group (7.00%) than in the slow freezing group (13.29%). The lower cooling rate used in the slow freezing protocol may have allowed a more gradual osmotic adjustment during cooling, thereby limiting abrupt changes in cell volume and membrane stress and contributing to the numerically higher HOST+ value observed after thawing. This interpretation should be considered cautiously because HOST+ values also differed significantly between the experimental groups before freezing. Therefore, the post-thaw difference in HOST+ cannot be attributed exclusively to the freezing protocol. Nevertheless, extenders that do not adequately meet the biochemical requirements of *C. porcellus* spermatozoa may compromise membrane integrity during this adaptation process.

Previous studies have reported HOST+ values of 74.97% in refrigerated spermatozoa [12], 60.49% in fresh epididymal spermatozoa [10], and 18.00% in sperm refrigerated at 5 °C [17], highlighting the considerable variability in plasma membrane functionality according to semen source and preservation conditions. Following thawing, a HOST+ value of 6.01% was reported using a Tris-based extender supplemented with 25% egg yolk [12], which was lower than the values obtained in the present study. Both refrigeration and cryopreservation substantially reduce the endosmotic capacity of the sperm plasma membrane [28]. Previous studies have suggested that extender composition and equilibration time may influence the preservation of plasma membrane functionality during cryopreservation [29]. Spermatozoa of *C. porcellus* appear to be more sensitive to temperature fluctuations than those of other mammalian species, probably due to their distinctive morphological characteristics [30].

Chromatin integrity was also assessed using toluidine blue staining (TB+) [31]. Following the six-hour equilibration period, condensed chromatin was observed in (84.76%) and (85.76%) of spermatozoa, with no significant differences between the experimental groups. After thawing, chromatin integrity decreased to (78.53%) in the rapid freezing protocol, whereas spermatozoa cryopreserved using the slow freezing protocol maintained a higher proportion of condensed chromatin (85.67%). These findings suggest that a gradual cooling rate may better preserve chromatin organization during cryopreservation by allowing a more gradual cellular response to cooling and reducing the structural stress associated with abrupt temperature changes. Consequently, the slow freezing protocol preserved post-thaw chromatin integrity more effectively than the rapid freezing protocol, supporting its potential to maintain sperm structural quality after thawing.

Cryopreservation differentially affected the morphometric characteristics of epididymal spermatozoa from *Cavia porcellus* depending on the cooling rate applied. The morphometric alterations were more pronounced following the rapid freezing protocol, whereas the slow freezing protocol largely preserved sperm morphometric characteristics after thawing. Specifically, rapid freezing induced significant reductions in acrosomal area, acrosomal perimeter, and sperm head length, whereas most nuclear, midpiece, and tail dimensions remained unchanged. In contrast, slow freezing preserved the majority of the morphometric variables evaluated, with only a significant reduction observed in midpiece width. This pattern suggests that the more gradual reduction in temperature allowed greater cellular adaptation to the physical changes accompanying cooling, potentially limiting abrupt osmotic and mechanical alterations that can affect sperm dimensions. These findings suggest that a gradual decrease in temperature may limit cryopreservation-induced structural alterations and contributes to the preservation of sperm morphology.

The greater susceptibility of the acrosome observed following rapid freezing may be attributed to its complex structural organization and membrane composition. During cooling and ice formation, sperm membranes undergo lipid phase transitions, osmotic imbalance, and mechanical stress, which may result in cell volume loss, membrane protein reorganization, and destabilization of the acrosomal matrix. Because the acrosome represents the outermost structure of the sperm head, it is particularly susceptible to cryoinjury and is often among the first sperm structures to undergo structural damage during freezing and thawing. In the present study, the significant reductions in acrosomal area and perimeter observed after rapid freezing are consistent with greater sensitivity of this compartment to the abrupt changes associated with the higher cooling rate. Conversely, the slower reduction in temperature may have allowed a more progressive adaptation of membrane water balance and lipid organization, potentially reducing structural deformation of the acrosomal region during cooling and thawing.

These findings are consistent with reports in several domestic mammalian species, in which cryopreservation preferentially affects the plasma membrane and acrosome before other sperm structures [32]. During cooling and thawing, membrane phospholipid phase transitions, osmotic stress, and ice crystal formation may induce physical and biochemical alterations that compromise acrosomal membrane stability and may compromise its structural integrity [33]. Ref. [34] demonstrated that cryopreservation induces changes resembling sperm capacitation, commonly referred to as cryocapacitation, which is characterized by membrane protein reorganization, lipid redistribution, and increased plasma membrane permeability. These alterations may predispose spermatozoa to acrosomal damage. In agreement with these mechanisms, rapid freezing in the present study resulted in a significant reduction in acrosomal area and perimeter, suggesting that this structure is particularly susceptible to abrupt temperature decline. Ref. [35] identified acrosomal integrity as one of the most sensitive indicators of sperm survival following cryopreservation, since damage to this structure compromises capacitation, the acrosome reaction, and ultimately fertilizing ability. From a biological perspective, the numerically greater preservation of acrosomal dimensions observed after slow freezing may reflect a more gradual adaptation of sperm membranes to cooling, allowing cellular water redistribution to occur progressively and potentially reducing abrupt osmotic changes and membrane deformation. In contrast, the higher cooling rate during rapid freezing may expose spermatozoa to more abrupt osmotic changes over a shorter period, potentially increasing membrane-associated stress and contributing to the greater acrosomal alteration observed in the present study. Cryopreservation may also promote oxidative damage to membrane lipids and proteins, which could further compromise membrane stability and acrosomal integrity. However, oxidative stress was not directly evaluated in the present study; therefore, this mechanism should be considered a plausible explanation for the observed differences rather than a demonstrated effect.

Correlation analysis also revealed associations among sperm morphometric traits after cryopreservation. Rapid freezing was characterized by fewer significant correlations and a predominance of negative associations between morphometric variables measured before freezing and after thawing. By comparison, slow freezing was associated with a greater number of positive correlations, particularly among nuclear dimensions, the midpiece, and other sperm head structures. The greater number of positive correlations observed after slow freezing, together with the limited number of negative associations, indicates the pattern of associations among morphometric traits after thawing.

The greater number of positive correlations observed after slow freezing was consistent with greater maintenance of associations among sperm morphometric traits after cryopreservation. Previous studies have shown that gradual cooling promotes more controlled cellular dehydration, reduces intracellular ice crystal formation, and facilitates the progressive redistribution of intracellular water [36]. These processes may reduce the mechanical and osmotic stresses generated during cooling.

Conversely, rapid freezing was characterized by negative associations, particularly between nuclear and acrosomal variables, suggesting that different sperm compartments do not respond uniformly to cryogenic stress. These alterations may reflect changes in the relationships among sperm compartments following cryopreservation. However, correlation analysis alone does not establish structural preservation or a causal effect of the freezing rate. This type of analysis has rarely been explored in *Cavia porcellus* spermatozoa; the present study suggests that morphometric correlation analysis may provide complementary information on the association among sperm morphometric traits that are not captured by conventional morphometric evaluation alone. However, the correlation coefficients were estimated from relatively small numbers of animals (*n* = 5 for rapid freezing and *n* = 7 for slow freezing), which limits the precision and generalizability of these associations. Therefore, the correlation patterns should be considered exploratory and interpreted cautiously, particularly given the limited sample size.

The absence of a split-sample design also represents a limitation, as the slow and rapid freezing protocols were applied to spermatozoa from different individuals, introducing potential interindividual variability that may affect the interpretation of the comparative results. Therefore, differences observed between freezing protocols cannot be attributed exclusively to the freezing treatment, and the causal interpretation of the protocol effect should be considered with caution.

The relatively small sample size is another limitation that should be considered when interpreting the present findings. Because no a priori power analysis was performed, the statistical power of the study to detect small or moderate differences between freezing protocols cannot be established with certainty. Consequently, the possibility of a Type II error cannot be excluded, particularly for outcomes in which statistically significant differences were not detected. Therefore, the absence of statistical significance should not be interpreted as evidence of equivalence between the freezing protocols. Studies including larger sample sizes will be necessary to increase statistical power and provide more robust estimates of the comparative effects of the freezing protocols. Overall, the findings of this study suggest that slow freezing was associated with greater numerical preservation of selected morphometric dimensions from a biological perspective.

## 5. Conclusions

Cryopreservation of *C. porcellus* spermatozoa remains challenging due to the high sensitivity of this species to cooling and freezing stress. The present study showed that slow freezing was associated with greater numerical preservation of selected morphometric dimensions and higher post-thaw chromatin integrity than rapid freezing, whereas several other sperm quality parameters did not differ significantly between protocols. Morphometric evaluation also showed fewer alterations after slow freezing, particularly in comparison with the changes observed following rapid freezing. In addition, characterization of the cooling curves using linear and cubic polynomial regression models provides a useful framework for optimizing and standardizing freezing protocols in this species. Furthermore, morphometric correlation analysis provided exploratory evidence of associations among sperm morphometric characteristics after thawing; however, these associations should be interpreted cautiously given the limited sample size. Overall, the findings suggest that slow freezing may be a promising approach for preserving selected structural characteristics of *C. porcellus* epididymal spermatozoa. However, this potential advantage should be interpreted cautiously because of the unequal sample sizes between freezing protocols, baseline differences between experimental groups, and the absence of a split-sample design. In addition, because the present study evaluated epididymal spermatozoa, the findings cannot be directly generalized to ejaculated semen. Further studies using balanced sample sizes, a split-sample design, and ejaculated spermatozoa are needed to determine the relative effects of freezing rate and their reproductive relevance.

## Figures and Tables

**Figure 1:**
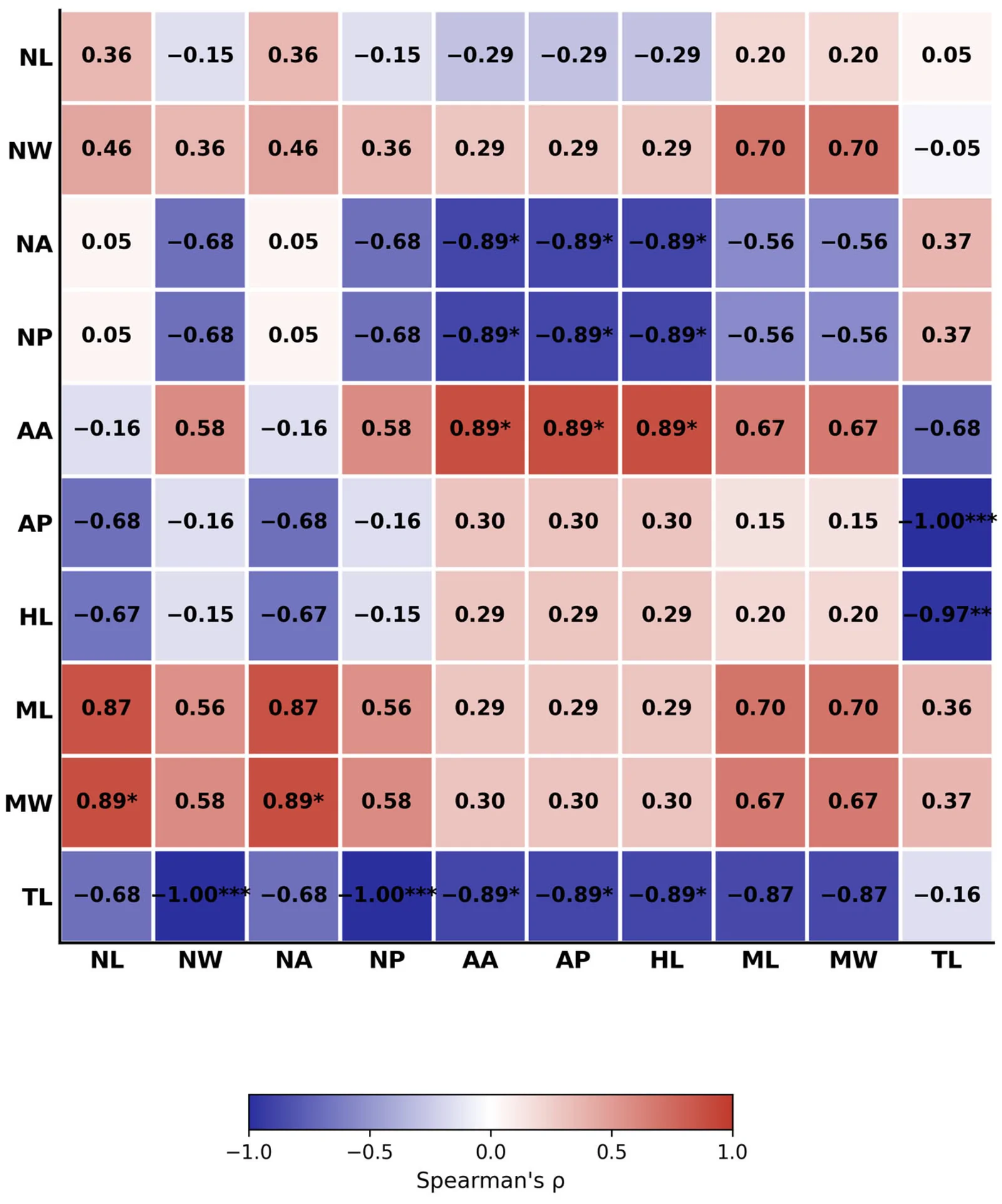
Heatmap illustrating Spearman’s correlation coefficients (ρ) between pre-freezing and post-thaw sperm morphometric variables following the rapid freezing protocol. Colors represent the strength and direction of the correlations, ranging from negative (blue) to positive (red). Significant correlations after Benjamini–Hochberg false discovery rate (FDR) correction are indicated by asterisks (* *p* < 0.05, ** *p* < 0.01, *** *p* < 0.001). Abbreviations: NL, nucleus length (µm); NW, nucleus width (µm); NA, nucleus area (µm^2^); NP, nucleus perimeter (µm); AA, acrosome area (µm^2^); AP, acrosome perimeter (µm); HL, head length (µm); ML, midpiece length (µm); MW, midpiece width (µm); TL, tail length (µm).

**Figure 2:**
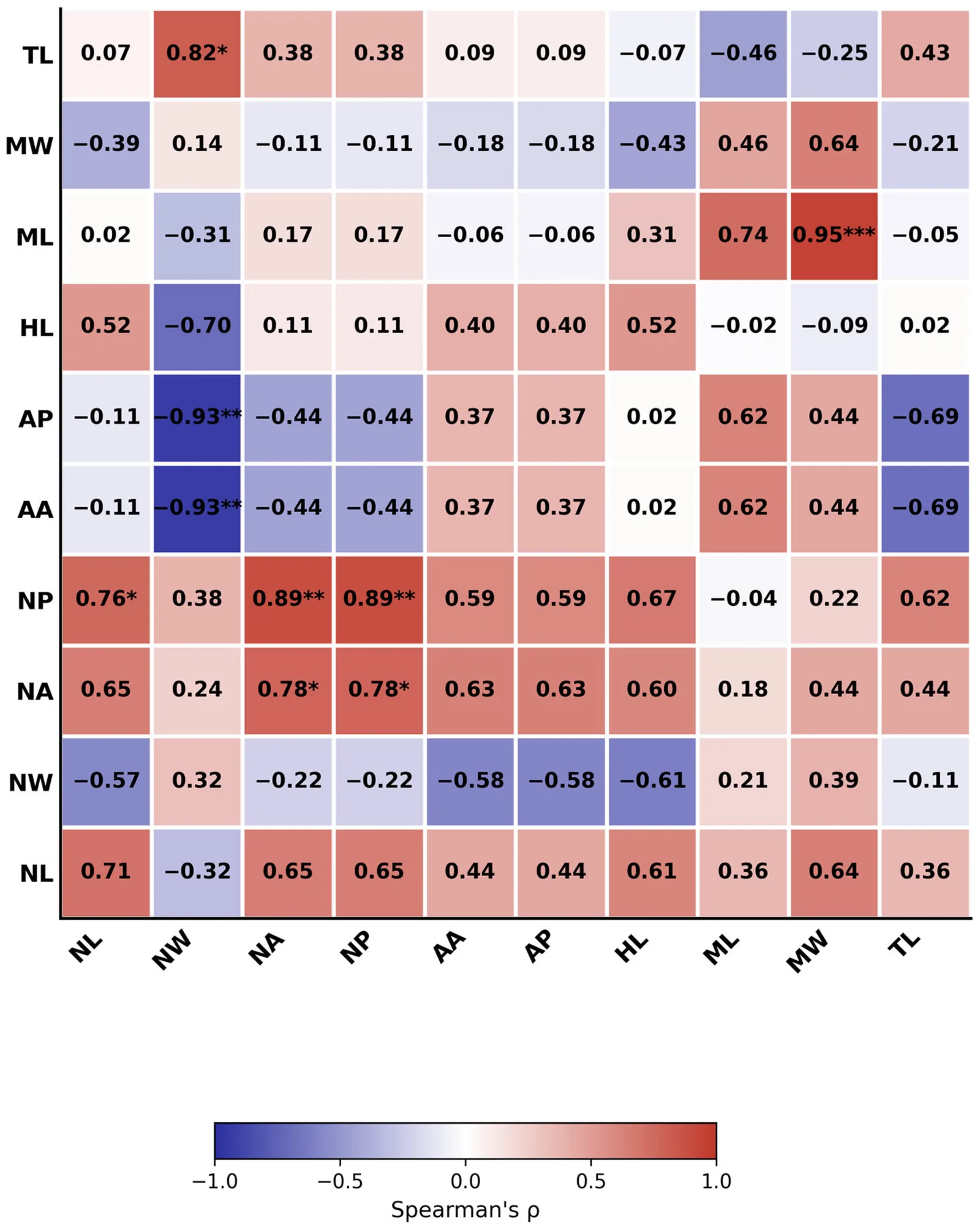
Heatmap illustrating the Spearman rank correlation coefficients (ρ) between sperm morphometric variables measured before cryopreservation (pre-freezing) and after thawing following the slow freezing protocol. Colors represent the strength and direction of the correlations, ranging from negative (blue) to positive (red). Significant correlations after Benjamini–Hochberg false discovery rate (FDR) correction are indicated by asterisks (* *p* < 0.05, ** *p* < 0.01, *** *p* < 0.001). Abbreviations: NL, nucleus length (µm); NW, nucleus width (µm); NA, nucleus area (µm^2^); NP, nucleus perimeter (µm); AA, acrosome area (µm^2^); AP, acrosome perimeter (µm); HL, head length (µm); ML, midpiece length (µm); MW, midpiece width (µm); TL (µm), tail length (µm).

**Figure 3:**
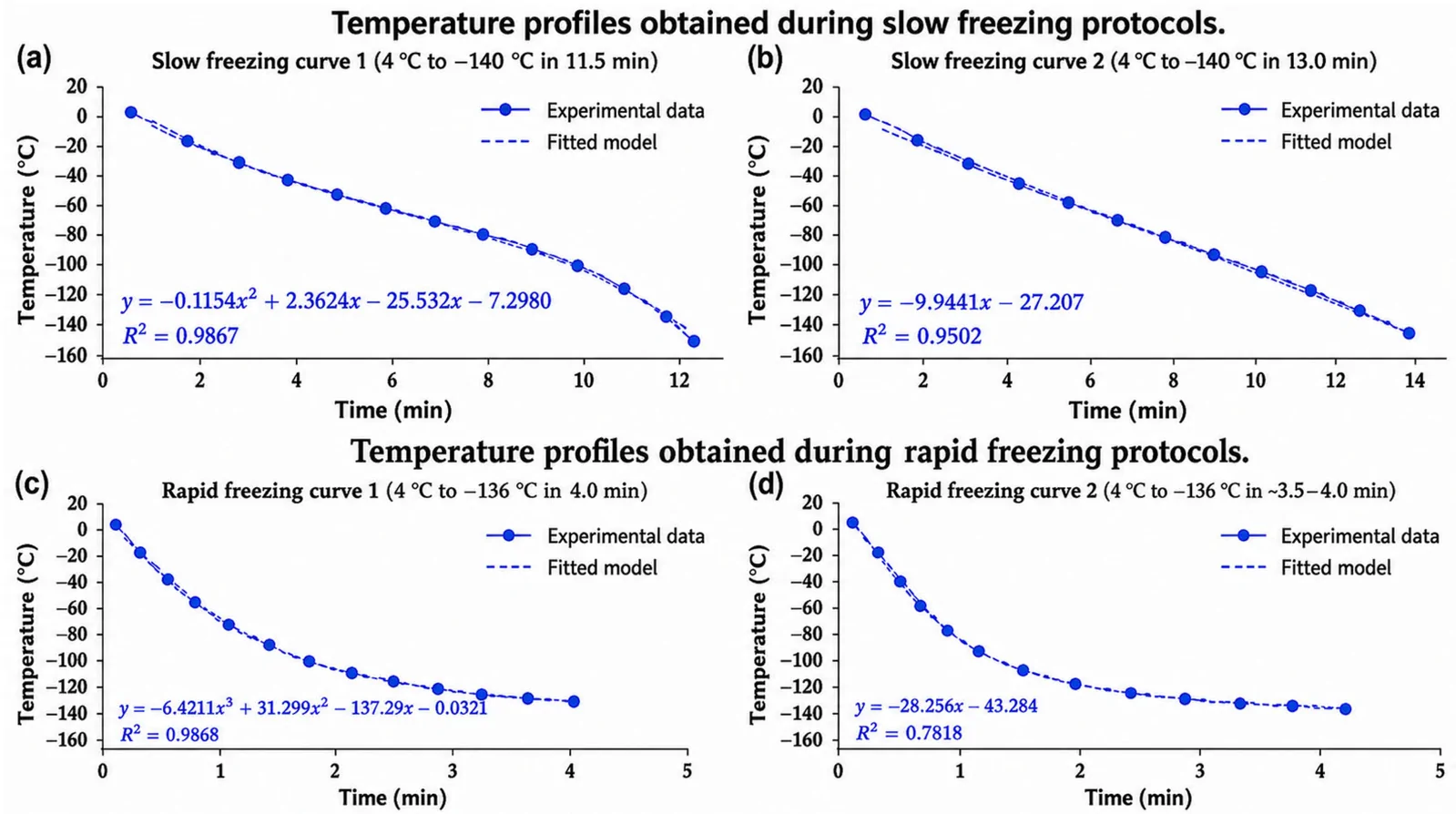
Slow (**a**,**b**) and rapid (**c**,**d**) freezing curves evaluated in the cryopreservation of *C. porcellus* spermatozoa.

**Table 1:** Comparison of microscopic characteristics of epididymal spermatozoa before freezing and after thawing following rapid and slow cryopreservation protocols.

Condition	N	Microscopic Characteristics (%) Mean ± SD				
		PM	Vitality	Acrosome	HOST+	Chromatin
Pre-freezing	5	65.07 ± 6.57	63.50 ± 8.58	46.88 ± 2.49	30.30 ± 7.65	84.73 ± 1.30
Rapid thawing	5	8.53 ± 1.74	15.70 ± 11.43	11.76 ± 6.59	7.00 ± 6.10	78.53 ± 3.98
Mean difference		56.53	47.8	35.12	23.3	6.2
95%CI		48.57–64.50	28.53–67.07	24.14–46.10	10.56–36.04	1.67–10.73
Cohen’s dz		8.81	3.08	3.97	2.27	1.7
*p*-value		0.00004	0.002	0.001	0.007	0.019
Pre-freezing	7	76.33 ± 7.20	69.71 ± 4.29	53.09 ± 12.81	23.71 ± 5.51	85.76 ± 7.36
Slow thawing	7	10.57 ± 2.14	18.36 ± 6.22	15.14 ± 5.64	13.29 ± 5.16	85.67 ± 5.79
Mean difference		65.76	51.36	37.94	10.43	0.1
95%CI		58.38–73.14	42.67–60.04	28.82–47.06	4.30–16.56	−3.35–3.54
Cohen’s dz		8.24	5.47	3.85	1.57	0.03
*p*-value		<0.001	<0.001	<0.001	0.006	0.948
Post-thaw comparison (Slow–Rapid)						
Mean difference		2.04	2.66	3.38	6.29	7.13
95% CI		−0.48–4.55	−11.29–16.60	−5.03–11.80	−1.49–14.06	0.85–13.42
Hedges’ g		0.95	0.28	0.52	1.04	1.28
*p*-value		0.10	0.65	0.38	0.10	0.03

Note: Data are presented as mean ± SD (standard deviation). N: number of *Cavia porcellus* males included in each cryopreservation protocol (rapid freezing, *n* = 5; slow freezing, *n* = 7), with one cryopreserved sample analyzed per animal. PM: progressive motility; HOST+: positive response to the hypo-osmotic swelling test. For the rapid and slow freezing groups, mean differences represent the within-animal difference between pre-freezing and post-thaw values. For the post-thaw comparison, mean differences were calculated as slow freezing minus rapid freezing. Cohen’s dz represents the standardized effect size for paired within-animal comparisons, whereas Hedges’ g represents the standardized effect size for the independent post-thaw comparison between freezing protocols. Differences were considered statistically significant at *p* < 0.05.

**Table 2:** Morphometric characteristics of epididymal spermatozoa before freezing and after thawing following rapid and slow cryopreservation protocols.

Morphometric Variables	Rapid Freezing				Slow Freezing			
	Pre-Freezing (Mean ± SD)	Post-Thaw (Mean ± SD)	*p*_Rapid	Effect Size	Pre-Freezing (Mean ± SD)	Post-Thaw (Mean ± SD)	*p*_Slow	Effect Size
NL (µm)	8.42 ± 0.27	8.56 ± 0.15	0.187	0.71 dz	8.35 ± 0.32	8.47 ± 0.23	0.123	0.68 dz
NW (µm)	7.71 ± 0.06	7.67 ± 0.15	0.628	−0.24 dz	7.51 ± 0.19	7.43 ± 0.22	0.256	−0.47 dz
NA (µm^2^)	54.17 ± 2.1	53.21 ± 1.91	0.547	−0.29 dz	52.72 ± 2.26	52.49 ± 1.29	0.681	−0.16 dz
NP (µm)	27.78 ± 0.59	27.57 ± 0.52	0.646	−0.22 dz	27.51 ± 0.53	27.42 ± 0.41	0.444	−0.31 dz
AA (µm^2^)	86.25 ± 5.89	59.57 ± 6.45	<0.001	−5.73 dz	87.11 ± 4.48	83.82 ± 5.05	0.25	−0.50 rrb
AP (µm)	35.87 ± 1.26	30.68 ± 1.36	<0.001	−4.23 dz	35.68 ± 1.14	35.34 ± 0.88	0.375	−0.43 rrb
HL (µm)	12.72 ± 0.38	11.68 ± 0.16	0.002	−3.06 dz	12.41 ± 0.44	12.26 ± 0.48	0.297	−0.50 rrb
ML (µm)	12.32 ± 0.74	13.86 ± 2.39	0.063	1.00 rrb	12.72 ± 1.69	12.44 ± 1.5	0.062	−0.86 dz
MW (µm)	0.52 ± 0.02	0.5 ± 0.03	0.051	−1.23 dz	0.5 ± 0.04	0.47 ± 0.03	0.039	−0.99 dz
TL (µm)	90.98 ± 1.38	84.8 ± 2.1	0.063	−1.00 rrb	89.7 ± 2.34	89.84 ± 2.16	0.777	0.11 dz

Note: Data are presented as mean ± SD (standard deviation). *p*-values were obtained using paired Student’s *t*-tests or Wilcoxon signed-rank tests, according to data distribution. Wilcoxon signed-rank tests were applied to midpiece length and tail length in the rapid freezing group and to acrosome area, acrosome perimeter, and head length in the slow freezing group. Effect sizes are reported as Cohen’s dz for paired Student’s *t*-tests and rank-biserial correlation (rrb) for Wilcoxon signed-rank tests. Differences were considered statistically significant (*p* < 0.05). Abbreviations: NL, nucleus length; NW, nucleus width; NA, nucleus area; NP, nucleus perimeter; AA, acrosome area; AP, acrosome perimeter; HL, head length; ML, midpiece length; MW, midpiece width; TL, tail length.

**Table 3:** Spearman correlation coefficients between pre-freezing and post-thaw sperm morphometric variables following the rapid freezing protocol.

Pre-Freezing	Rapid Freezing									
	NL (µm)	NW (µm)	NA (µm^2^)	NP (µm)	AA (µm^2^)	AP (µm)	HL (µm)	ML (µm)	MW (µm)	TL (µm)
NL (µm)	0.36	−0.15	0.36	−0.15	−0.29	−0.29	−0.29	0.20	0.20	0.05
NW (µm)	0.46	0.36	0.46	0.36	0.29	0.29	0.29	0.70	0.70	−0.05
NA (µm^2^)	0.05	−0.68	0.05	−0.68	−0.89 *	−0.89 *	−0.89 *	−0.56	−0.56	0.37
NP (µm)	0.05	−0.68	0.05	−0.68	−0.89 *	−0.89 *	−0.89 *	−0.56	−0.56	0.37
AA (µm^2^)	−0.16	0.58	−0.16	0.58	0.89 *	0.89 *	0.89 *	0.67	0.67	−0.68
AP (µm)	−0.68	−0.16	−0.68	−0.16	0.30	0.30	0.30	0.15	0.15	−1.00 ***
HL (µm)	−0.67	−0.15	−0.67	−0.15	0.29	0.29	0.29	0.20	0.20	−0.97 **
ML (µm)	0.87	0.56	0.87	0.56	0.29	0.29	0.29	0.70	0.70	0.36
MW (µm)	0.89 *	0.58	0.89 *	0.58	0.30	0.30	0.30	0.67	0.67	0.37
TL (µm)	−0.68	−1.00 ***	−0.68	−1.00 ***	−0.89 *	−0.89 *	−0.89 *	−0.87	−0.87	−0.16

Note: Spearman’s rank correlation coefficients (ρ) between pre-freezing and post-thaw sperm morphometric variables following the rapid freezing protocol. Statistical significance is indicated as * *p* < 0.05, ** *p* < 0.01, and *** *p* < 0.001 after Benjamini–Hochberg false discovery rate (FDR) correction. Abbreviations: NL, nucleus length; NW, nucleus width; NA, nucleus area; NP, nucleus perimeter; AA, acrosome area; AP, acrosome perimeter; HL, head length; ML, midpiece length; MW, midpiece width; TL, tail length.

**Table 4:** Spearman correlation coefficients between pre-freezing and post-thaw sperm morphometric variables following the slow freezing protocol.

Pre-Freezing	Slow Freezing									
	NL (µm)	NW (µm)	NA (µm^2^)	NP (µm)	AA (µm^2^)	AP (µm)	HL (µm)	ML (µm)	MW (µm)	TL (µm)
NL (µm)	0.71	−0.32	0.65	0.65	0.44	0.44	0.61	0.36	0.64	0.36
NW (µm)	−0.57	0.32	−0.22	−0.22	−0.58	−0.58	−0.61	0.21	0.39	−0.11
NA (µm^2^)	0.65	0.24	0.78 *	0.78 *	0.63	0.63	0.60	0.18	0.44	0.44
NP (µm)	0.76 *	0.38	0.89 **	0.89 **	0.59	0.59	0.67	−0.04	0.22	0.62
AA (µm^2^)	−0.11	−0.93 **	−0.44	−0.44	0.37	0.37	0.02	0.62	0.44	−0.69
AP (µm)	−0.11	−0.93 **	−0.44	−0.44	0.37	0.37	0.02	0.62	0.44	−0.69
HL (µm)	0.52	−0.70	0.11	0.11	0.40	0.40	0.52	−0.02	−0.09	0.02
ML (µm)	0.02	−0.31	0.17	0.17	−0.06	−0.06	0.31	0.74	0.95 ***	−0.05
MW (µm)	−0.39	0.14	−0.11	−0.11	−0.18	−0.18	−0.43	0.46	0.64	−0.21
TL (µm)	0.07	0.82 *	0.38	0.38	0.09	0.09	−0.07	−0.46	−0.25	0.43

Note: Spearman’s rank correlation coefficients (ρ) between pre-freezing and post-thaw sperm morphometric variables following the slow freezing protocol. Statistical significance was determined after Benjamini–Hochberg false discovery rate (FDR) correction and is indicated as * *p* < 0.05, ** *p* < 0.01, and *** *p* < 0.001. Abbreviations: NL, nucleus length; NW, nucleus width; NA, nucleus area; NP, nucleus perimeter; AA, acrosome area; AP, acrosome perimeter; HL, head length; ML, midpiece length; MW, midpiece width; TL, tail length.

**Table 5:** Summary of regression models fitted to representative cooling curves during slow and rapid cryopreservation of *Cavia porcellus* spermatozoa.

Curve	Freezing Rate	Protocol	Temperature Range (°C)	Time (min)	Regression Model	Equation	R^2^
(a)	Slow	Slow freezing curve 1	4–140	11.5	Cubic polynomial	*y* = −0.1154*x*^3^ + 2.5624*x*^2^ − 25.532*x* − 7.2983	0.9867
(b)	Slow	Slow freezing curve 2	4–140	13.0	Linear	*y* = −9.9444*x* − 27.207	0.9502
(c)	Rapid	Rapid freezing curve 1	4–136	4.0	Cubic polynomial	*y* = −6.4211*x*^3^ + 51.259*x*^2^ − 137.29*x* − 0.0323	0.9868
(d)	Rapid	Rapid freezing curve 2	4–136	~3.5–4.0	Linear	*y* = −28.256*x* − 43.224	0.738

Note: Representative cooling curves obtained during the slow and rapid cryopreservation protocols. Temperature profiles were modeled using linear or cubic polynomial regression according to the model that best fitted the data. Model fit is expressed as the coefficient of determination (R^2^).

## Data Availability

The data presented in this study are available from the corresponding author upon reasonable request.

## Abbreviations

The following abbreviations are used in this manuscript:

CB	Coomassie Blue
HOST	Hypoosmotic swelling test
HOST+	Positive hypoosmotic swelling reaction
TB	Toluidine Blue
TB+	Positive toluidine blue staining
CASA	Computer-assisted sperm analysis
